# Calcium-Involved Action of Phytochemicals: Carotenoids and Monoterpenes in the Brain

**DOI:** 10.3390/ijms21041428

**Published:** 2020-02-20

**Authors:** Jowita Rzajew, Tomasz Radzik, Elzbieta Rebas

**Affiliations:** Department of Molecular Neurochemistry, Medical University of Lodz, 92-216 Lodz, Polandtomasz.radzik@umed.lodz.pl (T.R.)

**Keywords:** calcium, NMDA, VGCC, carotenoids, monoterpenes, retinoic acid, neuroprotection

## Abstract

Background: Neurodegenerative and mood disorders represent growing medical and social problems, many of which are provoked by oxidative stress, disruption in the metabolism of various neurotransmitters, and disturbances in calcium homeostasis. Biologically active plant compounds have been shown to exert a positive impact on the function of calcium in the central nervous system. Methods: The present paper reviews studies of naturally occurring terpenes and derivatives and the calcium-based aspects of their mechanisms of action, as these are known to act upon a number of targets linked to neurological prophylaxis and therapy. Results: Most of the studied phytochemicals possess anticancer, antioxidative, anti-inflammatory, and neuroprotective properties, and these have been used to reduce the risk of or treat neurological diseases. Conclusion: The neuroprotective actions of some phytochemicals may employ mechanisms based on regulation of calcium homeostasis and should be considered as therapeutic agents.

## 1. Introduction

Phytochemicals are naturally occurring compounds in plants which, despite not being essential for the normal functioning of humans, have been found to exert several positive effects in animal cells; furthermore, they offer various health benefits and reduce the risk of various diseases when included in the diet. They are typically divided into several groups based on their chemical structure: polyphenols (with antioxidative, anticancerogenic, antibacterial, anti-inflammatory, and antithrombotic properties), carotenoids and mono- or diterpenes (antioxidative, anticancerogenic, and anti-inflammatory action), phytoestrogens (antioxidative and anticancerogenic properties), anthocyanins (antioxidative, anticancerogenic, and antithrombotic action), sulfides (antioxidative, anticancerogenic, antibacterial, anti-inflammatory, and antithrombotic action), glucosinolates (anti-inflammatory and antibacterial properties), saponins (anti-inflammatory and anticancerogenic action), and alkaloids (affecting central nervous system (CNS) functions) [1,2,3]. These compounds are known to be involved in the maintenance of cholesterol or glucose blood level [1,2]. Some phytochemicals, mainly polyphenols or carotenoids, have been found to act against neurodegenerative diseases like Alzheimer’s disease, Parkinson’s disease, Huntington’s disease, dementia, or various mood and affective diseases [4,5], possibly by modifying the metabolism and action of neurotransmitters [5,6]. Some studies indicate that they also act by modulating calcium signaling. Although some alkaloids are strong toxins, most phytochemicals are considered safe and the risk of overdosing is very low; even so, greater knowledge about their effects on various processes in the central nervous system is needed due to their growing application in drugs and dietary supplements.

In brain cells and neurons, calcium acts as an important intracellular messenger participating in the regulation of growth, differentiation, exocytosis, synaptic transmission, and the processes of learning and memory [7,8]. Disturbances in calcium homeostasis can lead to various neurodegenerative and psychological disorders or brain aging [8]. Low calcium concentration is tightly maintained in the cytosol by multiple mechanisms based around the regulation of calcium influx/efflux by ion channels: NMDA receptors (NMDAR) [8,9], AMPA/kainate receptors (AMPAR) [10], voltage-gated calcium channels (VGCCs) [11], Na^+^/Ca^2+^ exchangers (NCX) [11,12], ATP-dependent calcium pumps [13], G protein–coupled receptors (e.g., metabotropic glutamate receptor, mGluR) [14], IP_3_ receptors [15], transient receptor potential cation channels (TRP receptors) [16], or binding to buffer proteins (e.g., calbindins, parvalbumin) [17]. The presence of such a large number of calcium-dependent signaling pathways raises a range of questions regarding the calcium-engaged action of phytochemicals. The present paper reviews the activity of selected terpenes on cellular calcium signaling components and their potential application as neuroprotective agents.

## 2. The Effect of Terpenes and Their Derivatives on Calcium-Engaged Signaling

The terpenes, and their terpenoid derivatives, comprise a group of organic compounds synthesized by a variety of plants, flowers, and coniferous trees, as well as some animals. They often protect plants against disease or deter herbivores. Terpenes are built from isoprene units and are divided into several classes according to the numbers of units, some examples being hemiterpenes, sesquiterpenes, monoterpenes, diterpenes, tetraterpenes, and polyterpenes [18,19]. Some terpenoids are used by the human body to synthesize essential compounds, for example, vitamin A from β-carotene [18]. Most terpenoids exert various biological effects in animal organisms, including those associated with the nervous system, and although several terpenoids, such as those derived from *Cannabis*, have psychoactive properties, most have a positive influence on the nervous system [18,19]. The terpenes described in this review are listed in Table 1 [20,21,22,23,24,25,26].

### 2.1. Carotenoids

Carotenoids are natural pigments occurring in plants, fungi, bacteria, and algae. They are responsible for the red, orange, and yellow colours of flowers, ripe fruits, leaves, and roots, and the intensity of colour is generally correlated with the amount of carotenoids [27,28]. In plants they dissipate light energy not used for photosynthesis, such as UV, and protect against oxidative stress through their antioxidant properties [27]. Not being able to synthesise them themselves, animals obtain carotenoids from their diet. Interestingly, although more than 600 carotenoids are known to exist, fewer than 40 are present in the typical human diet [29]. Carotenoids consist of tetraterpenoid units formed of a C40 carbon skeleton and exist as long chains possessing alternating double and single bonds. The compounds are divided into polar xanthophylls, with oxygen as a functional group, and nonpolar carotenes, lacking a functional group. Carotenoids can be also classified as provitamin A and non-provitamin A [27,30].

Carotenoids are absorbed in a similar way to lipids and are differentially distributed throughout the human body. Sixteen carotenoids have been found within the human brain; of these, xanthophylls account for up to 77%. The localization of carotenoids within the cell depends on the functional groups which determine their hydrophobic nature: nonpolar carotenes are vertically integrated inside the membrane whereas polar xanthophylls cross the bilayer at a 40° slant [27].

There is evidence that carotenoids may play an important role in the prevention or in supporting the treatment of a range of diseases, many of which are related to oxidative stress, e.g., age-related macular disease, cancer, cardiovascular diseases, and neurodegenerative diseases [27,31]. They are thought to exert their neuroprotective potential through neutralization of reactive oxygen species (ROS) and other free radicals by stimulation of superoxide dismutase, catalase, and glutathione peroxidase (lutein, β-carotene, zeaxanthin, crocetin, crocin, astaxanthin, β-cryptoxanthin, lycopene); reduction of neuroinflammation by suppressing the activation of the nuclear factor κB pathway or lowering proinflammatory cytokine generation (lutein, astaxanthin, fucoxanthin, lycopene); and modulation of autophagy (crocin, lycopene) [27,28,32]. Some studies indicate that carotenoids can also modulate calcium signalling [27].

#### 2.1.1. Astaxanthin

Astaxanthin (AXT) is a xanthophyll found in many plants, types of seafood, and algae. It is an approved dietary supplement with the ability to cross the blood–brain barrier but without significant side-effects. Reports suggest that it demonstrates antihepatotoxicity, antitumor, anti*-Helicobacter pylori*, and anti-inflammatory properties [33,34]. It has much higher antioxidant potential than other carotenoids [33]. This compound can influence calcium homeostasis in various ways.

Excess levels of glutamate, the main excitatory neurotransmitter, can lead to overstimulation of ligand-dependent NMDA or AMPA channels and result in a postsynaptic influx of calcium up to cytotoxic levels. Any mechanism which decreases NMDA or AMPA receptor amount or activity is believed to protect against glutamate- and calcium-evoked excitotoxicity. Astaxanthin can inhibit the expression of NMDA receptor subunits in primary cortical neurons in a dose- and time-dependent manner. It was found that 24 h and 48 h exposure to 10 or 20 nM astaxanthin significantly reduced NR3A subunit expression, while a shorter, 4 h incubation prevented any further increase of expression. In addition, 24 h treatment with 20 nM astaxanthin reduced the mRNA expression of other subunits (NR1, NR2A, NR2B); a temporary increase was observed after 4 h exposure [35]. Although NR2A and NR2B should share similar molecular structures and functions, they do in fact display a number of structural differences in receptors with various subunit compositions [36]. The NR2B subunit is highly expressed in the prenatal stages and its expression drops at the postnatal stages, becoming focally expressed in the forebrain; in contrast, NR2A is expressed at low levels in the prenatal stages and increases upon birth. Additionally, variation in *GRIN2A* (the gene for the NR2A subunit) is commonly associated with an epileptic phenotype, while that in *GRIN2B* (the gene for the NR2B subunit) is commonly found in patients with neurodevelopmental disorders [36]. The distinct effect of astaxanthin on various NMDA receptor subunits may be significant in facilitating prolonged neuroprotection against high glutamate levels in people with neurological or psychiatric disorders.

As Ca^2+^ influx plays an important role in pain signaling by enhancing neurotransmitter release and altering cell membrane excitability, excessive NMDARs activity can result in the development of neuropathic pain. In silico molecular docking studies have shown that astaxanthin perfectly fits into the inhibitory binding pocket of NMDA receptors, particularly NR2B protein, which is involved in nociception. Astaxanthin may represent a potential alternative in the treatment of chronic neuropathic pain, possibly by inactivating NMDA receptors [37].

The neuroprotective properties of astaxanthin were highlighted in studies using differentiated PC12 cells treated with MPP+. MPP+ (n-methyl-4-phenylpyridinium iodide) is the toxic metabolite of 1-methyl-4-phenyl-1,2,3,6-tetrahydropyridine (MPTP), a well-established and commonly used substance used in the toxic model of Parkinson’s disease. In the presence of AXT, PC12 cell viability was significantly increased, and Sp1 (activated transcription factor-1) and NR1 decreased at the mRNA and protein levels compared to in the MPP+ groups without AXT [38].

AXT is also believed to reduce neurotoxicity in cell culture models of Alzheimer’s disease. One of the major hypotheses of the development of Alzheimer’s disease is the accumulation of β-amyloid (β-A) oligomers (β-AOs) [39]. Astaxanthin can protect cells against β-amyloid toxicity by downregulation of apoptotic factors, inhibition of proinflammatory cytokine activity action, and reduction of ROS [27]. AXT exposure is known to reduce amyloid-β-induced generation of ROS and calcium dysregulation in primary hippocampal neurons. Results suggest that ATX protects neurons from the noxious effects which β-amyloid exerts on mitochondrial ROS production, NFATc4 activation, and downregulation of RyR2 gene expression. Six-hour incubation with β-A (500 nM) significantly decreased RyR2 mRNA levels to approximately 54%. Preincubation with ATX (0.10 µM) did not modify RyR2 mRNA levels but blocked the reduction of RyR2 mRNA levels promoted by β-amyloid. Incubation of primary hippocampal neurons with AβOs results in significant downregulation of RyR2 mRNA and protein levels; it is possible that these reductions are crucial to the synaptotoxicity induced by β-A. Of note, postmortem samples of patients who died with AD display significantly reduced RyR2 expression at early stages of the disease [40].

Astaxanthin also affects the mRNA expression of L-type voltage-gated calcium channels (L-VGCC) in a dose-, channel-type-, and time-dependent way in post-synaptic primary cortical neurons. After 4 h treatment with 20 nM AXT, only L-VGCC A1D-type mRNA expression was increased; however, prolonged incubation up to 48 h had no effect. L-VGCC A1C expression was decreased by 20 nM AXT after four hours, but both 10 nM and 20 nM concentrations of AXT caused stimulation of expression after 48 h. Increased amounts of both types of L-VGCC and downstream of calcium-induced depolarization stimulate calcium-dependent non-specific ion channels or calcium-dependent potassium channels. Calcium influx through L-VGCC regulates calcium signaling pathways, including activation of CREB (cAMP response element-binding protein). Differential modulation of L-VGCC by astaxanthin can play a role in the maintenance of calcium homeostasis in cells [35].

Additional mechanisms exist by which astaxanthin can protect cells against glutamate cytotoxicity. AXT inhibited 4-aminopyridine (4-AP)-evoked release of glutamate in rat cerebral cortex in a dose-dependent manner. This effect was blocked by chelating intrasynaptosomal Ca^2+^ ions and by treatment with vesicular transporter inhibitor and N-, P-, and Q-type Ca^2+^ channel blockers; however, treatment with glutamate transporter inhibitors, ryanodine receptor blockers, or mitochondrial Na^+^/Ca^2+^ exchanger blockers had no effect. AXT also was found to decrease calcium gains induced by depolarization. The inhibitory effect of astaxanthin on glutamate release was prevented by mitogen-activated protein kinase (MAPK) inhibitors PD98059 and U0126. The results indicated that astaxanthin inhibits glutamate release from rat cortical synaptosomes through the suppression of presynaptic voltage-dependent calcium entry and the MAPK signaling cascade [41].

Astaxanthin can also modify calcium homeostasis by increasing the mRNA level of calbindin D28k and parvalbumin, two buffering proteins which decrease the total amount of free cytosolic Ca^2+^ by binding cytoplasmatic calcium ions. This effect was observed after 48 h of treatment with 10 nM astaxanthin [35].

Some of the enzymes involved in calcium signaling pathways can be modified by astaxanthin. Calpains are cytosolic calcium-dependent cysteine proteases. While they remain inactivated in the absence of Ca^2+^, elevation of intracellular calcium levels results in calpain overactivation and, thus, detrimental effects on neurons: abnormally high activity promotes apoptotic processes, neurodegeneration, and, ultimately, neuronal death [42]. In vitro studies indicate that astaxanthin protects porcine lens proteins from hydrolysis by calpains; the results suggest that this carotenoid reacts with calcium ions, decreases the free Ca^2+^ level, and thus mitigates the probability of calpain activation [34,43].

Calcineurin (CaN) is a calcium- and calmodulin-dependent serine/threonine protein phosphatase found abundantly in the CNS. It can affect the basal excitability of neurons by modulating the function of many ion channels, including L-type Ca^2+^ channels, potassium channels, and voltage-gated sodium (Na^+^) channels via their dephosphorylation. Moreover, reports suggest that it inhibits presynaptic glutamate release. CaN has also been shown to enhance or prolong desensitization of ionotropic receptors like NMDA, γ-aminobutyric acid, serotonin, and acetylcholine receptors. Calcineurin plays an important role in a number of processes by dephosphorylation of a number of proteins, one example being nuclear factor of activated T-cells (NFAT). Plaques of amyloid beta seem to activate CaN. Calcineurin is subsequently involved in Alzheimer’s disease, indirectly mediating such events as tau hyperphosphorylation, synaptic plasticity, and neurotransmission decrease or neuroinflammation [44]. During the prolonged Ca^2+^ signaling evoked in Alzheimer’s disease, the isoform NFATc4 is activated by CaN, resulting in such abnormalities as neuritic dystrophy and loss of dendritic branching and spines. *In vitro* research on hippocampal neurons indicates that preincubation with AXT prevents NFATc4 activation by β-A and CaN [40].

A study comparing the neuroprotective properties of astaxanthin with those of β-carotene and canthaxanthin using undifferentiated PC12 cells found that AXT increased cell viability and partially inhibited H_2_O_2_-induced cytotoxicity at 0.5–5.0 µM, while β-carotene and canthaxanthin provided little or no cell protection against H_2_O_2_. Astaxanthin significantly inhibited β-amyloid (25–35)-induced cytotoxicity at 0.5–10.0 µM, but canthaxanthin and β-carotene exerted medium or low inhibition of β-amyloid (25–35)-induced cytotoxicity. These findings suggest that the neuroprotective activity exhibited by astaxanthin is due to its ·O_2_^−^, ·OH, and H_2_O_2_ scavenging activities, and the presence of hydroxyl and ketone groups in its structure. Canthaxanthin possesses only ketone groups but β-carotene does not have any hydroxyl or ketone groups in its structure; both are considered to have lower antioxidant activity than astaxanthin. All three compounds are known to decrease intracellular calcium levels to a similar degree. The influx of calcium into undifferentiated PC12 cells was significantly enhanced by H_2_O_2_ and β-amyloid (25–35) and is clearly reduced by co-incubation with 10 µM astaxanthin. The high activity of astaxanthin in decreasing calcium production is highly dependent on its antioxidant activity and on its neuroprotective activity against β-amyloid (25–35) toxicity to undifferentiated PC12 cells. Both β-carotene and canthaxanthin are able to modulate calcium production, but their action is weaker than that of AXT [45].

#### 2.1.2. Lycopene

Lycopene is one of the most famous carotenoids and is found in many red and orange plants, including tomatoes, watermelons, apricots, grapefruits, and guavas. It is well known for its strong antioxidant properties and is believed to prevent cardiovascular diseases, decrease blood pressure in patients who suffer from hypertension, demonstrate anticancer properties, inhibit cyclin D and E expression, and possibly invoke suppression of the cell cycle in phase G1. There has been some evidence that lycopene exhibits a neuroprotective profile in the central nervous system, but few studies have rigorously explored the role of neurotransmitters in this regard [46].

It was reported that lycopene (0.1–5 µM) inhibited 4-aminopyridine (4-AP)-evoked glutamate release in a dose-dependent manner in rat cerebrocortical nerve terminals. The Cav2.2 (N-type) and Cav2.1 (P/Q-type) channel blocker ω-conotoxin-MVIIC reduced the inhibitory effect of lycopene, whereas the intracellular calcium-release inhibitors dantrolene and CGP37157 had no effect. Furthermore, treatment with protein kinase C inhibitors GF109203X and Go6976, blocked the action of lycopene on glutamate release. These results suggest that lycopene inhibits glutamate release from rat cortical synaptosomes by suppressing presynaptic Ca^2+^ entry and protein kinase C activity [47].

Intracellular calcium homeostasis may be disturbed by neurotoxins. Methylmercury (MeHg), an organic form of mercury, may be a good example. MeHg affects the CNS, resulting in serious disorders like cerebellar ataxia, paresthesia, dysarthria, mnemic deficits, and memory impairment, as well as various sensory disorders. It invokes a wide range of molecular impairments, including disruption of Ca^2+^ homeostasis. It is believed that MeHg promotes the elevation of intracellular calcium via glutamate-mediated excitotoxicity or targeting mitochondrial calcium stores. If sustained, this elevation may lead to many molecular events resulting in cell death [48,49]. Experiments conducted on cultured rat cerebellar granule neurons indicate that lycopene (0.5–10 µM) effectively prevents the loss of cell viability induced by MeHg. Pretreatment with lycopene also inhibited induced mitochondrial oxidative stress and mitochondrial respiratory dysfunction in cells, as well as reductions in mtDNA content and mtDNA transcript levels [49].

Cadmium (Cd) is a well-known, highly toxic environmental contaminant. It can cause neurotoxicity by modulating autophagy, affecting ion homeostasis, and triggering redox stress. Cd has been shown to induce autophagy in mouse brains. It has been also shown that Cd exposure inhibits the activities of Ca^2+^ -ATPase and Ca^2+^/Mg^2+^-ATPase in mouse hippocampus, resulting in abnormally high intracellular Ca^2+^ concentrations. Lycopene (5 mg/kg) blocked both these phenomena, restoring the intracellular Ca^2+^ concentration to control levels and increasing Ca^2+^-ATPase and Ca^2+^/Mg^2+^-ATPase activity. Lycopene treatment also increased the expression of several Ca^2+^-ATPase isoforms downregulated by Cd [50].

#### 2.1.3. β-Carotene

β-carotene is an orange-red lipid-soluble precursor to vitamin A. It is found in vegetables and fruits, and it is sometimes used as food coloring. It is known for its antioxidant properties. Some studies also indicate its anti-cancer activities [51].

Calcium/calmodulin-dependent protein kinase IV (CAMKIV) is an enzyme belonging to the Ser/Thr kinase family. In the brain, it is found in the cerebellar cortical granules. It plays a role in cell proliferation, migration, angiogenesis, inhibition of apoptosis, and cell signaling in a calcium-dependent manner. At elevated intracellular calcium ion concentration, CAMKIV forms Ca^2+^/calmodulin complexes [52] and induces the phosphorylation of transcription factors. CAMKIV is considered an important factor in neurodegenerative disorders and various types of cancer. It has been shown that β-carotene is able to bind to the active site of CAMKIV with high affinity, thus forming a stable complex, resulting in decreased CAMKIV activity. This ability could make β-carotene an attractive target in cancer treatment, since it has shown no cytotoxic effects *in vitro* [53].

#### 2.1.4. Crocin and Crocetin

Crocin and its aglycone crocetin are water-soluble carotenoids present in *Crocus sativus*, commonly known as saffron. The plant is applied in traditional medicine for the treatment of cramps, asthma, fever, edema, hepatic disorders, and depression, among others. Crocin is also used as a seasoning or coloring agent in food [54]. The compounds in saffron, mainly crocin and crocetin, have been reported to have antiproliferative, antitumor, anti-inflammatory, antioxidant, antiapoptotic, and hepatoprotective effects [54,55].

It was observed that *Crocus sativus* extracts (CSE) (10–200 μg/mL) inhibited postsynaptic potentials (PSPs), elevated the isolated NMDA and non-NMDA components of PSPs, and decreased glutamate-induced membrane depolarization in rat cortical brain slices. *Trans*-crocetin alone (1–50 μM) reduced evoked PSPs and glutamate-induced membrane depolarization similarly to CSE. Treatment with 10 μM *trans*-crocetin decreased NMDA-induced membrane depolarization, but did not affect the isolated non-NMDA components of PSPs; therefore, the authors suggest that this carotenoid may be involved in the modulation of NMDA receptors but not AMPA/kainate receptors [56].

Other *in vivo* and *in vitro* studies have found that crocin abolishes the inhibitory effect of ethanol on long-term potentiation (LTP) in rat hippocampus. As 10 µM crocin significantly blocked the inhibition of NMDA response by 10–50 mM ethanol but did not affect the inhibition of non-NMDA response by 100 mM ethanol, it has been proposed that this mechanism probably involves the NMDA receptor [57].

Works on the interaction between saffron-derived compounds and NMDA receptor binding sites suggest that CSE and crocetin bind to the PCP binding site of the NMDA receptor and at the sigma-1 receptor, while the crocins and picrocrocin do not [58].

**Figure 1:**
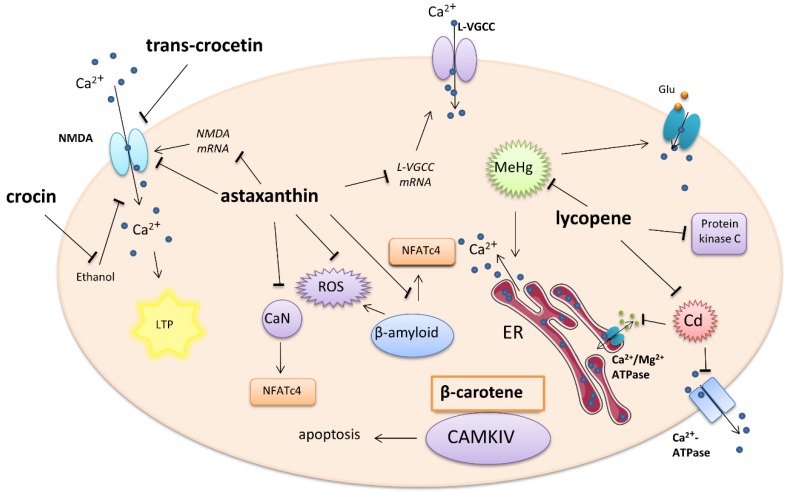
Effect of carotenoids on intracellular calcium-involved actions. Arrows—stimulation or calcium influx/efflux; T-bars—inhibition of pathway; ER—endoplasmatic reticulum, Glu—glutamate

A summary of tetraterpene–carotenoid activity on calcium-involved cellular signaling is presented in Figure 1.

### 2.2. Retinoic Acid (Diterpene)

Retinoic acid (RA), one of the active forms of vitamin A, is a derivative of pre-vitamin A β-carotene and a direct metabolite of vitamin A_1_ (all-*trans*-retinol). Structurally, retinoic acid is a diterpene. It is an important agent in the development and regeneration of the nervous system and modulation of hippocampal synaptic plasticity, such as long-term potentiation and depression. Three isoforms of retinoic acid are described: all-*trans*-retinoic acid (ATRA), 9-*cis*-retinoic acid, and 13-*cis*-retinoic acid. Retinoic acid can act in a non-genomic manner; however, it mostly exerts its properties through the activation of two specific receptors: retinoic acid receptors (RAR) and retinoid X receptors (RXR). The resulting retinoid acid–receptor complex generally acts as a transcriptional factor and influences gene expression, which then determines genomic action [59,60].

Calcium homeostasis can be modulated by RA through both genomic and non-genomic routes. It has been shown that ATRA can affect both calcium channel expression and intracellular calcium concentration in a genomic way [61]. RA has previously been shown to alter the expression of L- and N-type VGCCs during RA-mediated neuronal differentiation [61].

**Figure 2:**
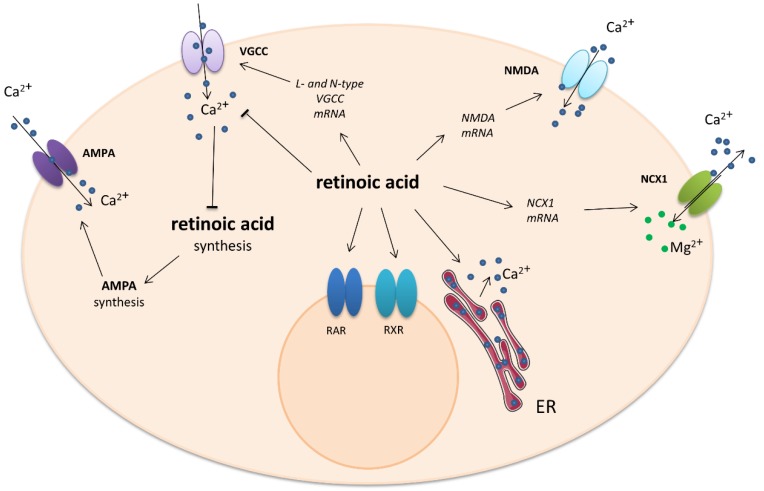
Impact of retinoic acid on intracellular calcium-involved actions. Arrows—stimulation or calcium influx/efflux; T-bars—inhibition of pathway. ER—endoplasmatic reticulum

One non-genomic effect involves the modulation of Ca^2+^ levels during homeostatic synaptic plasticity in the hippocampus. Exposure to RA resulted in a rapid decrease in intracellular calcium concentration in a manner dependent on dose and isoform. A significant decrease in calcium was observed from 15 min after application of 10 µM ATRA until 60 min. No reduction in intracellular calcium was observed following exposure to a lower (1 µM) concentration of ATRA or 10 µM 9-*cis*-RA. The ATRA-induced decrease in calcium level was not blocked by the RA receptor antagonists LE540 for RAR and HX531 for RXR. In addition, ATRA was not mimicked by the calcium-dependent potassium channel inhibitor Apamin. Also, it was shown that ATRA can reduce transmembrane calcium influx through the VGCCs of adult neurons [61]. The Na^+^/Ca^2+^ exchanger is an important calcium channel which regulates calcium levels by the exchange of three Na^+^ ions for one of Ca^2+^ and can act in either direction. Three mammalian types of Na^+^/Ca^2+^ exchanger are expressed in the neuronal tissue: NCX1, NCX2, and NCX3. It was reported that RA increased the levels of NCX1 mRNA in the rat brain stem and hypothalamus but not the cerebellum [62]. Similar results were obtained using cultured invertebrate motor neurons. Overnight exposure to physiological concentrations of RA significantly inhibited the voltage-gated Ca^2+^ current in an isomer-dependent manner. ATRA depolarized the voltage of half-maximal activation of I_Ca_, but 9-*cis*-RA did not. ATRA also reduced the rate of channel activation and delayed recovery from inactivation. Because both nifedipine-sensitive and nifedipine-resistant I_Ca_ were inhibited in these neurons, it is most likely that both L-type and non-L-type voltage-gated Ca^2+^ channels are affected by ATRA. These effects of retinoic acid are thought to be at least partially mediated by the retinoid receptors, as treatment of the neurons with synthetic RAR and RXR agonists produced similar inhibition of I_Ca._ [63]. The action of retinoic acid on calcium-engaged signaling is shown in Figure 2.

The study showed that retinoic acid may play a role in regulating the expression of the NR1 subunit of the NMDA receptor. The levels of RARα, Src, and NR1 in the neurons of prenatal marginal vitamin A deficiency (mVAD) rats, in terms of both mRNA and protein level, were significantly lower than those of healthy rats, resulting in impaired learning and memory. It was also found that mVAD neurons induced by RA treatment displayed a lower increase in RARα, Src, and NR1 expression than normal neurons. Additionally, RARα, Src, and NR1 expression levels were also elevated in normal neurons with overexpression of the RARα gene [64].

A relationship has been found between intracellular calcium level and RA synthesis, with RA synthesis being activated when postsynaptic Ca^2+^ entry is significantly decreased. The fact that calcium influx is blocked by stimulation of RA synthesis by synaptic glutamate receptor (AMPAR) or L-type VGCC treatment suggests that the source of calcium entry does not play a significant role in the suppression of RA synthesis. The increase of all-*trans*-retinoic acid in turn increases AMPA receptor synthesis. In conclusion, modest “basal” levels of postsynaptic calcium physiologically suppress RA synthesis in synaptically active neurons, whereas decreases in the resting calcium levels induce homeostatic plasticity in synaptically inactive neurons by stimulating RA synthesis, which then acts in a cell-autonomous manner to increase AMPA receptor function [65]

### 2.3. Monoterpenes

#### 2.3.1. Linalool

Linalool, a monoterpene alcohol, is present as a main component in the essential oils of certain aromatic plant species, including *Lavandula angustifolia, Melissa officinalis*, and *Rosmarinus officinalis.* It is used as a sedative, analgesic, anti-inflammatory, anxiolytic, and antiepileptic agent in traditional medicine thanks to its antioxidative properties. It is suggested that linalool plays an important role in protection against neurodegenerative diseases because it is able to reduce the amyloid plaques and tau hyperphosphorylation in Alzheimer’s disease (mouse model), with improvements in behavioral assessments of learning and memory [66,67,68].

At a low concentration (0.1 mM), linalool induces suppressive and antiepileptic activity that is probably mediated by its inhibition of the Na^+^ inward current and by indirect potentiation of Ca^2+^-activated K^+^ currents. However, higher concentrations of linalool (0.4 mM) elicited excitatory and epileptogenic effects that seemed to be mediated by inhibitory action on K^+^ channels and were dependent on Ca^2+^ inward currents and PKC activity. The linalool-induced epileptiform activity was abolished by Ca^2+^ channel blockers, nifedipine and nickel chloride, and selective inhibitor of protein kinase C, chelerythrine [66].

It was observed that co-treatment with 100 μM linalool reduced the increase in mitochondrial Ca^2+^ levels caused by 18 h glutamate exposure in HT-22 cells; however, higher concentrations of linalool (200–500 µM) exerted an opposite effect. Mitochondria play an essential role in buffering against cytosolic calcium overload in stimulated neurons. Obtained data indicate that linalool may be therapeutically useful in treating neurodegenerative conditions marked by mitochondrial dysfunction and subsequent ROS production [67,68].

Linalool is one of the monoterpenes found in bergamot essential oil (BEO). It was reported that BEO can prevent the accumulation of calpain-specific 150–145 kDa spectrin breakdown products caused by five-minute exposure of SH-SY5Y cells to NMDA, indicating that inhibition of calpain activation may play a role in neuroprotection. In addition to the activation of Ca^2+^-dependent enzymes, such as nitrogen reactive species (NOS) and calpain I, calcium entry through the NMDA receptor-gated channel triggers mitochondrial Ca^2+^ overload and dysfunction leading to ROS generation and, ultimately, to cell death [69].

#### 2.3.2. Thymol

Another monoterpene which exerts antioxidative, anti-inflammatory, antinociceptive, and vasorelaxant properties is thymol; it is mainly found in the common culinary herbs thyme and oregano. Additionally, thymol is used as a flavoring agent in the food industry. Thymol exerts protective effects against rotenone-induced neurodegeneration in a rat model of Parkinson’s disease [70]; it has also been found to reduce Aβ plaques and improve memory in a high-fat-diet-fed rat model of Alzheimer’s disease [71].

Like other monoterpenes, the action of thymol is concentration dependent. It is known to release Ca^2+^ from intracellular stores in different cell types, including neurons [72]. It has been found that 400 μM thymol induces transient calcium release from internal stores, and a higher concentration (1 mM) produces steady Ca^2+^ leakage in neurons of the snail (*Helix pomatia*) [73]. Calcium channel blockers abolish thymol-induced burst firing. The application of NiCl, T-type VGCC inhibitor, or nifedipine, an L-type VGCC blocker, gradually suppressed thymol (1 mM)-induced burst firing and silenced the neurons of snail *Caucasotachea atrolabiata* [74]. Thymol (1 mM) altered action potential characteristics and provoked epileptiform burst firing in snail neurons [74].

#### 2.3.3. Paeoniflorin

In recent years, paeoniflorin, a monoterpene glycoside isolated from peony root, has been widely studied as an antioxidant, anticonvulsant, neuroprotective, antithrombotic, and antidepressant agent, as well as a cognitive enhancer or learning impairment attenuating agent. Treatment of PC12 cells with glutamate for 24 h caused a significant increase in the concentration of intracellular Ca^2+^ and a decrease in the Calbindin-D28K mRNA. Pre-incubation of glutamate-treated PC12 cells with paeoniflorin (1, 10, and 50 mM) for two hours resulted in the concentration of intracellular Ca^2+^ significantly decreasing in a dose-dependent manner as compared with the glutamate group. The same concentrations of paeoniflorin also significantly increased Calbindin-D28K mRNA levels in glutamate-treated PC12 cells. The results suggest that paeoniflorin exerts a neuroprotective effect on glutamate-induced neurotoxicity in PC12 cells, at least in part via inhibiting Ca^2+^ overload and the following ROS generation [75].

**Figure 3:**
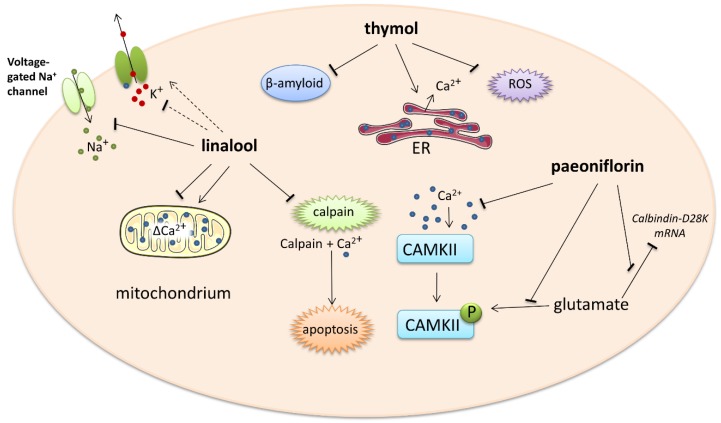
Impact of monoterpenes on intracellular calcium-involved actions. Arrows—stimulation or calcium influx/efflux; T-bars—inhibition of pathway; dotted line—indirect influence. ER—endoplasmatic reticulum, ROS—reactive oxygen species

The next experiment showed that the intracellular calcium level and phosphorylation of calcium/calmodulin-dependent protein kinase II (CAMKII) significantly increased in differentiated PC12 cells exposed to glutamate compared to untreated cells. This glutamate-induced phosphorylation and intracellular calcium overload was suppressed by 100 μM paeoniflorin in a time-dependent manner. Excessive intracellular Ca^2+^ may induce neuronal cell injury and death via activation of the CaMKII signaling pathway since paeoniflorin appears to be a neuroprotective agent. Albiflorin, an isomer of paeoniflorin also found in peony root, did not exert any action against glutamate-evoked changes in calcium homeostasis. The improvement of cell damage by the CaMKII inhibitor KN93 further confirms the role of CaMKII in paeoniflorin-mediated neuroprotection [76]. The action of monoterpenes is shown in more detail in Figure 3.

## 3. Conclusions

Many studies confirm that terpenes possess neuroprotective properties. These can be expressed through various mechanisms, such as the reduction of ROS generation, modulation of neurotransmitter metabolism or action, and inhibition of apoptosis or prevention of β-amyloid accumulation. As indicated by the studies above, some mechanisms involve the modification of calcium homeostasis, especially with regard to changing the total amount of calcium and the activity of the calcium channels. The action of terpenes depends on their type, concentration, and time of exposure. Carotenoids are known to have positive effects on the CNS, and the most active carotenoids, astaxanthin and lycopene, are still safe at higher concentrations. The activity of di- and monoterpenes generally depends on their doses.

The blood levels of selected terpenes after administration of a single dose and their effective concentrations which exert biological effects are presented in Table 2 [77,78,79,80,81,82,83,84,85,86]. In most cases a higher single dose is required to reach the effective terpene concentration in the serum; however, the half time of these compounds’ elimination from the blood is rather long, and repeated doses or a daily diet containing these compounds may be sufficient to maintain a safe, but stable, effective concentration.

Although a great deal of work has already been performed on the potential of phytochemicals in medicine, further studies of the action of terpenes are required. Nevertheless, it is undoubtedly beneficial for human health to include carotenoids in the daily diet.

## Figures and Tables

**Table 1:** Classification, structures, and action of terpenes and terpenoids described in this article.

Class of Terpenes	Name of Compound	Plant Source	Structure	Affected Component of Calcium Signaling
Tetraterpenes -xantophylles	Astaxanthin	Species in the genus *Adonis*	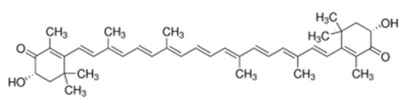	↓NMDAR expression ↑RYR2 expression ↓L-VGCC A1D expression ↓VGCC current ↑Calbindin expression ↑Parvalbumin expression ↓calpain activity ↓calcineurin activity ↓glutamate toxicity
Tetraterpene β-carotenes	Lycopene	Tomatoes, watermelons, pink grapefruits, apricots, pink guavas	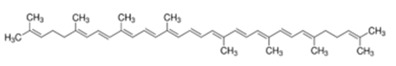	↓Cav2.1 activity ↓Cav2.2 activity ↓glutamate toxicity ↓ PKC activity ↑Ca/ATPase expression
	β-carotene	Carrots, spinach, pumpkins, papayas, sweet potatoes, winter squash, mangoes, cantaloupes, red peppers	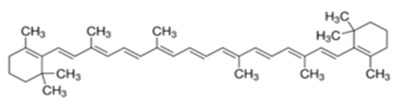	↓CAMKIV activity
	Crocin and crocetin	*Crocus sativus*, Gardenia	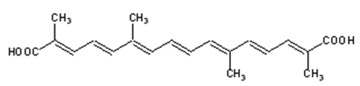	↓NMDAR current ↓glutamate toxicity
Diterpenes	Retinoic acid	Mangos, papayas, many of the squashes, carrots, sweet potatoes *	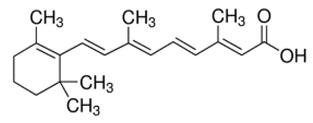	L-VGCC expression N-VGCC expression VGCC activity ↑NCX1 expression ↓AMPAR expression
Monoterpenes	Linalool	*Lauraceae,* Citrus fruits, Birch trees	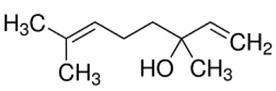	↓↑Ca-dependent K-channel PKC activity ↓calpain
	Thymol	Thyme, Origanum, Ajowan	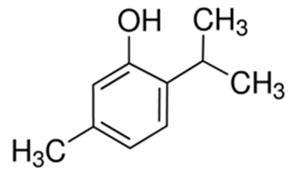	L-VGCC action T-VGCC action
	Paeoniflorin	Paeonia lactiflora	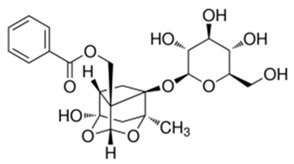	↓glutamate toxicity ↑calbindin expression ↓CaMKII activity

* sources for precursor of retinoic acid—β-carotene.

**Table 2:** Comparison of serum concentrations after administration of a single dose of selected terpenes and effective concentrations which exert biological effects for some exemplary compounds.

	Single Dose	Peak Level *	Time to Reach Peak Level	Elimination Half Time	Effective Concentration/dose **	Target
Astaxanthin	100 mg	1.3 µg/mL (2.17 µM) ^#^	7 h	21 ± 11 h	10–20 nM 100 nM 20 nM 0.5–10 µM	NMDAR subunits RAR VGCC Ca^2+^ level
Lycopene from tomato sauces	4 mg or 13 mg	30 nM or 0.2 µM	5 h	> 6 h	0.1–5 µM 0.5–10 µM 5 mg/kg **	Cav2.2, Cav2.1 Glutamate excitotoxicity Ca^2+^-ATPase
Lycopene from papaya	13 mg	20–30 nM	6 h	> 6 h		
Lycopene supplement	15 mg	40 nM	10 h	Lack of data		
β-carotene from tomato sauces	17 mg	70 nM	5 h	> 6 h	18.6–186 µM	CaMKIV
β-carotene from papaya	13 mg	17–28 nM	6 h	> 6 h		
Crocetin	Tea from 200 mg saffron	4 µM	2 h	Lack of data	1–50 µM	NMDAR
Crocetin-supplement	25 µg	400 ng/mL (1.2 µM) ^#^	1.5 h	90 min		
Retinoic acid-supplement	40 mg	83 ng/mL (0.28 µM) ^#^	3 h	6 h	10 µM	VGCC
Linalool-transdermally		100–150 ng/mL (0.65–0.97 µM) ^#^	0.3 h	1 h	0.1–0.4 mM	K^+^ current
Thymol-supplement	1.08 mg	93 ng/mL (0,62 µM) ^#^	1.9 h	10 h	1 mM	VGCC
Paeoniflorin	5 mg	6 ng/mL (12.5 nM) ^#^	3 h	1.73	1–50 mM or 100 µM	↓ Ca^2+^i Or CaMKII

* The highest concentration measured in blood serum. ** One single dose (mg/kg of body weight) after administration of which a biological effect was detected. ^#^ own calculation. ↓ decrease of calcium level

## Abbreviations

NMDAR	N-methyl-D-aspartate receptor
AMPAR	α-amino-3-hydroxy-5-methyl-4-isoxazolepropionic acid receptor
VGCC	Voltage-gated calcium channel
NCX	Sodium–Calcium Exchanger
RyR	Ryanodine receptor
PKC	Protein kinase C
CAMK	Calcium/calmodulin-dependent kinase type II or IV
AXT	Astaxanthin
RA	Retinoic acid
ATRA	All-*trans*-retinoic acids
RAR	Retinoic acid receptor
RXR	Retinoid X receptors

## References

[1] Charu G., Dhan P. (2014). Phytonutrients as therapeutic agent. J. Complement. Integr. Med..

[2] Liu R.H. (2013). Health-promoting components of fruits and vegetables in the diet. Adv. Nutr..

[3] Hussain G., Rasul A., Anwar H., Aziz N., Razzaq A., Wei W., Ali M., Li J., Li X. (2018). Role of Plant Derived Alkaloids and Their Mechanism in Neurodegenerative Disorders. Int. J. Biol. Sci..

[4] Bhullar K.S., Rupasinghe H.P. (2013). Polyphenols: Multipotent therapeutic agents in neurodegenerative diseases. Oxid. Med. Cell Longev..

[5] Panche A.N., Diwan A.D., Chandra S.R. (2016). Flavonoids: An overview. J. Nutr. Sci..

[6] Rebas E., Rzajew J., Radzik T., Zylinska L. (2020). Neuroprotective Polyphenols: A Modulatory Action on Neurotransmitter Pathways. Curr. Neuropharmacol..

[7] Zündorf G., Reiser G. (2011). Calcium Dysregulation and Homeostasis of Neural Calcium in the Molecular Mechanisms of Neurodegenerative Diseases Provide Multiple Targets for Neuroprotection. Antioxid. Redox Signal..

[8] Pchitskaya E., Popugaeva E., Bezprozvanny I. (2018). Calcium signaling and molecular mechanisms underlying neurodegenerative diseases. Cell Calcium..

[9] Guo H., Camargo L.M., Yeboah F., Digan M.E., Niu H., Pan Y., Reiling S., Soler-Llavina G., Weihofen W.A., Wang H.R. (2017). A NMDA-receptor calcium influx assay sensitive to stimulation by glutamate and glycine/D-serine. Sci. Rep..

[10] König N., Poluch S., Estabel J., Durand M., Drian M.J. (2001). Synaptic and Non-synaptic AMPA Receptors Permeable to Calcium. Jpn. J. Pharmacol..

[11] Gleichmann M., Mattson M.P. (2011). Neuronal Calcium Homeostasis and Dysregulation. Antioxid. Redox Signal..

[12] Bano D., Ankarcrona M. (2018). Beyond the critical point: An overview of excitotoxicity, calcium overload and the downstream consequences. Neurosci. Lett..

[13] Brini M., Carafoli E. (2009). Calcium Pumps in Health and Disease. Physiol. Rev..

[14] Niswender C.M., Conn J. (2010). Metabotropic Glutamate Receptors: Physiology, Pharmacology, and Disease. Annu. Rev. Pharmacol. Toxicol..

[15] Kania E., Roest G., Vervliet T., Parys J.B., Bultynck G. (2017). IP3 Receptor-Mediated Calcium Signaling and Its Role in Autophagy in Cancer. Front. Oncol..

[16] Armita S. (2018). Transient Receptor Potential (TRP) Channels. Subcell Biochem..

[17] Fairless R., Williams S.K., Diem R. (2019). Calcium-Binding Proteins as Determinants of Central Nervous System Neuronal Vulnerability to Disease. Int. J. Mol. Sci..

[18] Pichersky E., Raguso R.A. (2018). Why do plants produce so many terpenoid compounds?. New Phytologist..

[19] Tetali S.D. (2019). Terpenes and isoprenoids: A wealth of compounds for global use. Planta.

[20] Francis X., Cunningham E.G. (2011). Elucidation of the Pathway to Astaxanthin in the Flowers of *Adonis Aestivalis*. Plant Cell.

[21] Maoka T. (2020). Carotenoids as natural functional pigments. J. Nat. Med..

[22] Singla R.K., Varadaraj B.G. (2011). Crocin: An Overview. Indo Glob. J. Pharm. Sci..

[23] Sarkar A.K. (2019). Emerging Trends of Bioscience Research.

[24] Gilbert C. (2013). What is vitamin A and why do we need it?. Community Eye Health J..

[25] Sharma S., Anand N. (1997). Approaches to Design and Synthesis of Antiparasitic Drugs.

[26] Zhang L., Wei W. (2019). Anti-inflammatory and immunoregulatory effects of paeoniflorin and total glucosides of paeony. Pharm. Ther..

[27] Cho K.S., Shin M., Kim S., Lee S.B. (2018). Recent advances in studies on the therapeutic potential of dietary in neurodegenerative diseases. Oxid. Med. Cell Longev..

[28] Milani A., Basirnejad M., Shahbazi S., Bolhassani A. (2017). Carotenoids: Biochemistry, pharmacology and treatment. Br. J. Pharmacol..

[29] Sudhahar V., Fukai T., Tsukahara H., Kaneko K. (2014). Studies on Pediatric Disorders.

[30] Mezzomo N., Ferreira S.R.S. (2016). Carotenoids Functionality, Sources, and Processing by Supercritical Technology: A Review. J. Chem..

[31] Mohammadzadeh-Honarvar N., Saedisomeolia A., Abdolahi M., Shayeganrad A., Taheri-Sangsari G., Hassanzadeh-Rad B. (2017). Molecular Anti-inflammatory Mechanisms of Retinoids and Carotenoids in Alzheimer’s Disease: A Review of Current Evidence. J. Mol. Neurosci..

[32] Zhang L., Wang H. (2015). Multiple Mechanisms of Anti-Cancer Effects Exerted by Astaxanthin. Mar. Drugs.

[33] Grimmig B., Kim S.H., Nash K., Bickford P.C., Douglas S.R. (2017). Neuroprotective mechanisms of astaxanthin: A potential therapeutic role in preserving cognitive function in age and neurodegeneration. GeroScience.

[34] Wu T.H., Liao J.H., Hou W.C., Huang F.Y., Maher T.J., Hu C.C. (2006). Astaxanthin protects against oxidative stress and calcium-induced porcine lens protein degradation. J. Agric. Food Chem..

[35] Altunrende M.E., Gezen-Ak D., Atasoy I.L., Candas E., Dursun E. (2018). The Role of Astaxanthin on Transcriptional Regulation of NMDA receptors Voltage Sensitive Calcium Channels and Calcium Binding Proteins in Primary Cortical Neurons. Arch. Neuropsychiatry.

[36] Myers S.J., Yuan H., Kang J.Q., Tan F.C.K., Traynelis S.F., Low C.M. (2019). Distinct roles of *GRIN2A* and *GRIN2B* variants in neurological conditions. F1000Research.

[37] Sharma K., Sharma D., Sharma M., Sharma N., Bidve P., Prajapati N., Kalia K., Tiwari V. (2018). Astaxanthin ameliorates behavioral and biochemical alterations in in-vitro and in-vivo model of neuropathic pain. Neurosci. Lett..

[38] Ye Q., Zhang X., Huang B., Zhu Y., Chen X. (2013). Astaxanthin suppresses MPP(+)-induced oxidative damage in PC12 cells through a Sp1/NR1 signaling pathway. Mar. Drugs.

[39] Cline E.N., Bicca M.A., Viola K.L., Klein W.L. (2018). The Amyloid-Oligomer Hypothesis: Beginning of the Third Decade. J. Alzheimer’s Dis..

[40] Lobos P., Bruna B., Cordova A., Barattini P., Galáz J.L., Adasme T., Hidalgo C., Muñoz P., Paula-Lima A. (2016). Astaxanthin Protects Primary Hippocampal Neurons against Noxious Effects of Aβ-Oligomers. Neural. Plast..

[41] Lin T.Y., Lu C.W., Wang S.J. (2010). Astaxanthin inhibits glutamate release in rat cerebral cortex nerve terminals via suppression of voltage-dependent Ca(2+) entry and mitogen-activated protein kinase signaling pathway. J. Agric. Food Chem..

[42] Momeni H.R. (2011). Role of Calpain in Apoptosis. Cell J..

[43] Yildiz-Unal A., Korulu S., Karabay A. (2015). Neuroprotective strategies against calpain-mediated neurodegeneration. Neuropsychiatr. Dis. Treat..

[44] Reese C.L., Taglialatela G. (2011). A Role for Calcineurin in Alzheimers Disease. Curr. Neuropharmacol..

[45] Chang C.S., Chang C.L., Lai G.H. (2013). Reactive oxygen species scavenging activities in a chemiluminescence model and neuroprotection in rat pheochromocytoma cells by astaxanthin, beta-carotene, and canthaxanthin. Kaohsiung J. Med. Sci..

[46] Przybylska S. (2019). Lycopene—A bioactive carotenoid offering multiple health benefits: A review. IJFST.

[47] Lu C.W., Hung C.F., Jean W.H., Lin T.Y., Huang S.K., Wang S.J. (2018). Lycopene depresses glutamate release through inhibition of voltage-dependent Ca2+ entry and protein kinase C in rat cerebrocortical nerve terminals. Can. J. Physiol. Pharmacol..

[48] Roos D., Seeger R., Puntel R., Vargas Barbosa N. (2012). Role of calcium and mitochondria in MeHg-mediated cytotoxicity. J. Biomed. Biotechnol..

[49] Qu M., Nan X., Gao Z., Guo B., Liu B., Chen Z. (2013). Protective effects of lycopene against methylmercury-induced neurotoxicity in cultured rat cerebellar granule neurons. Brain Res..

[50] Zhang F., Xing S., Li Z. (2017). Antagonistic effects of lycopene on cadmium-induced hippocampal dysfunctions in autophagy, calcium homeostatis and redox. Oncotarget.

[51] Silalahi J. (2002). Anticancer and health protective properties of citrus fruit components. Asia Pac. J. Clin. Nutr..

[52] Naz H., Islam A., Ahmad F., Hassan M. (2016). Calcium/calmodulin-dependent protein kinase IV: A multifunctional enzyme and potential therapeutic target. Prog. Biophys. Mol. Biol..

[53] Naz H., Khan P., Tarique M., Rahman S., Meena A., Ahamad S., Luqman S., Islam A., Ahmad F., Hassan M.I. (2017). Binding studies and biological evaluation of β-carotene as a potential inhibitor of human calcium/calmodulin-dependent protein kinase IV. Int. J. Biol. Macromol..

[54] Suleria H.A.R., Barrow C. (2019). Crocin, a Mechanistic Treatise. Bioactive Compounds from Plant Origin: Extraction, Applications, and Potential Health Benefits.

[55] Yorgun M.A., Rashid K., Aslanidis A., Bresgen C., Dannhausen K., Langmann T. (2017). Crocin, a plant-derived carotenoid, modulates microglial reactivity. Biochem. Biophys. Rep..

[56] Berger F., Hensel A., Nieber K. (2011). Saffron extract and trans-crocetin inhibit glutamatergic synaptic transmission in rat cortical brain slices. Neuroscience.

[57] Abe K., Sugiura M., Shoyama Y., Saito H. (1998). Crocin antagonizes ethanol inhibition of NMDA receptor-mediated responses in rat hippocampal neurons. Brain Res..

[58] Lechtenberg M., Schepmann D., Niehues M., Hellenbrand N., Wünsch B., Hensel A. (2008). Quality and functionality of saffron: Quality control, species assortment and affinity of extract and isolated saffron compounds to NMDA and sigma1 (sigma-1) receptors. Planta Med..

[59] Zhang R., Wang Y., Li R., Chen G. (2015). Transcriptional Factors Mediating Retinoic Acid Signals in the Control of Energy Metabolism. Int. J. Mol. Sci..

[60] Napoli J.L., Race K.R. (1988). Biogenesis of retinoic acid from beta-carotene. Differences between the metabolism of beta-carotene and retinal. J. Biol. Chem..

[61] Vesprini N.D., Dawson T.F., Yuan Y., Bruce D., Spencer G.E. (2015). Retinoic acid affects calcium signaling in adult molluscan neurons. Neurophysiol.

[62] Hudecova S., Stefanik P., Macejova D., Brtko J., Krizanova O. (2004). Retinoic Acid Increased Expression of the Na+/Ca2+ Exchanger in the Heart and Brain. Gen. Physiol. Biophys..

[63] De Hoog E., Lukewich M.K., Spencer G.E. (2018). Retinoic acid inhibits neuronal voltage-gated calcium channels. Cell Calcium..

[64] Zhang X., Yuan X., Chen L., Wei H., Chen J., Li T.J. (2017). The change in retinoic acid receptor signaling induced by prenatal marginal vitamin A deficiency and its effects on learning and memory. Nutr. Biochem..

[65] Wang H.L., Zhang Z., Hintze M., Chen L. (2011). Decrease in Calcium Concentration Triggers Neuronal Retinoic Acid Synthesis during Homeostatic Synaptic Plasticity. J. Neurosci..

[66] Vatanparast J., Bazleh S., Janahmadi M. (2017). The effects of linalool on the excitability of central neurons of snail Caucasotachea atrolabiata. Comp. Biochem. Physiol..

[67] Elisabetsky E., Brum L.F., Souza D.O. (1999). Anticonvulsant properties of linalool in glutamate-related seizure models. Phytomedicine.

[68] Sabogal-Guáqueta A.M., Hobbie F., Keerthi A., Oun A., Kortholt A., Boddeke E., Dolga A. (2019). Linalool attenuates oxidative stress and mitochondrial dysfunction mediated by glutamate and NMDA toxicity. Biomed. Pharmacother..

[69] Corasaniti M.T., Maiuolo J., Maida S., Fratto V., Navarra M., Russo R., Amantea D., Morrone L.A., Bagetta G. (2007). Cell signaling pathways in the mechanisms of neuroprotection afforded by bergamot essential oil against NMDA-induced cell death in vitro. Br. J. Pharmacol..

[70] Javed H., Azimullah S., Meeran M.F.N., Ansari S.A. (2019). Neuroprotective effects of thymol, a dietary monoterpene against dopaminergic neurodegeneration in rotenone induced rat model of Parkinson’s disease. Int. J. Mol. Sci..

[71] Asadbegi M., Yaghmaei P., Salehi I., Komaki A., Ebrahim-Habibi A. (2017). Investigation of thymol effect on learning and memory impairment induced by intrahippocampal injection of amyloid beta peptide in high fat diet- fed rats. Metab. Brain Dis..

[72] Shen A.Y., Huang M.H., Wang T.S., Wu H.M., Kang Y.F., Chen C.L. (2009). Thymol evoked Ca^+^ mobilization and ion currents in pituitary GH3 cells. Nat. Prod. Commun..

[73] Kostyuk P.G., Belan P.V., Tepikin A.V. (1991). Free calcium transients and oscillations in nerve cells. Exp. Brain. Res..

[74] Zolfaghari Z., Vatanparast J. (2019). Thymol provokes burst of action potentials in neurons of snail Caucasotachea atrolabiata. Comp. Biochem. Physiol. C Toxicol. Pharmacol..

[75] Mao Q.Q., Zhong X.M., Feng H.R., Pan A.J., Li Z.Y., Huang Z. (2010). Protective Effects of Paeoniflorin Against Glutamate-Induced Neurotoxicity in PC12 Cells via Antioxidant Mechanisms and Ca^2+^ Antagonism. Cell Mol. Neurobiol..

[76] Wang D., Tan Q.R., Zhang Z.J. (2013). Neuroprotective Effects of peoniflorin, But Not the Isomer Albiflorin, are Associated with the Suppression of Intracellular Calcium and Calcium/Calmodulin Protein Kinase II in PC12 Cells. J. Mol. Neurosci..

[77] Østerlie M., Bjerkeng B., Liaaen-Jensen S. (2000). Plasma appearance and distribution of astaxanthin E/Z and R/S isomers in plasma lipoproteins of men after single dose administration of astaxanthin. J. Nutr. Biochem..

[78] Unlu N.Z., Bohn T., Francis D., Clinton S.K., Schwartz S.J. (2007). Carotenoid Absorption in Humans Consuming Tomato Sauces Obtained from Tangerine or High-*β*-Carotene Varieties of Tomatoes. J. Agric. Food. Chem..

[79] Schweiggert R.M., Kopec R.E., Villalobos-Gutierrez M.G., Hogel J., Quesada S., Esquivel P., Schwartz S.J., Carle R. (2014). Carotenoids are more bioavailable from papaya than from tomato and carrot in humans: A randomised cross-over study. Br. J. Nutr..

[80] Riso P., Brusamolino A., Contino D., Martini D., Vendrame S., DelBo’ C., Porrini M. (2010). Lycopene absorption in humans after the intake of two different single-dose lycopene formulations. Pharmacol. Res..

[81] Chryssanthi G.C., Lamari F.N., Georgakopoulos C.D., Cordopatis P. (2011). A new validated SPE-HPLC method for monitoring crocetin in human plasma—Application after saffron tea consumption. J. Pharm. Biomed. Anal..

[82] Almodóvar P., Briskey D., Rao A., Prodanov M., Inarejos-García A.M. (2020). Bioaccessibility and Pharmacokinetics of a Commercial Saffron (*Crocus sativus* L.) Extract. Hindawi Evid. Based Complementary Altern. Med..

[83] Schmitt-Hoffmann A.H., Roos B., Sauer J., Schleimer M., Kovācs P., Stoeckel K., Maares J. (2011). Influence of food on the pharmacokinetics of oral alitretinoin (9-cis retinoic acid). Clin. Exp. Dermatol..

[84] Jäger W.R., Buchbauer G., Jirovetz L., Fritzer M. (1992). Percutaneous absorption of lavender oil from a massage oil. J. Soc. Cosmet. Chem..

[85] Kohlert C., Schindler G., März R.W., Abel G., Brinkhaus B., Derendorf H., Gräfe E.U., Veit M. (2002). Systemic availability and pharmacokinetics of thymol in humans. J. Clin. Pharmacol..

[86] Sadakane C., Watanabe J., Fukutake M., Nisimura H., Maemura K., Kase Y., Kono T. (2015). Pharmacokinetic Profiles of Active Components After Oral Administration of a Kampo Medicine, Shakuyakukanzoto, to Healthy Adult Japanese Volunteers. J. Pharm. Sci..

